# Three Distinct Glutamate Decarboxylase Genes in Vertebrates

**DOI:** 10.1038/srep30507

**Published:** 2016-07-27

**Authors:** Brian P. Grone, Karen P. Maruska

**Affiliations:** 1 Department of Neurological Surgery, University of California San Francisco, San Francisco, CA, 94143, USA; 2 Department of Biological Sciences, Louisiana State University, Baton Rouge, LA, 70803, USA

## Abstract

Gamma-aminobutyric acid (GABA) is a widely conserved signaling molecule that in animals has been adapted as a neurotransmitter. GABA is synthesized from the amino acid glutamate by the action of glutamate decarboxylases (GADs). Two vertebrate genes, *GAD1* and *GAD2*, encode distinct GAD proteins: GAD67 and GAD65, respectively. We have identified a third vertebrate GAD gene, *GAD3*. This gene is conserved in fishes as well as tetrapods. We analyzed protein sequence, gene structure, synteny, and phylogenetics to identify *GAD3* as a homolog of *GAD1* and *GAD2*. Interestingly, we found that *GAD3* was lost in the hominid lineage. Because of the importance of GABA as a neurotransmitter, *GAD3* may play important roles in vertebrate nervous systems.

Glutamate decarboxylases (GADs) are essential for the conversion of glutamate to **γ**-aminobutyric acid (GABA), the predominant inhibitory neurotransmitter in central nervous systems^1^. GADs are members of the Group II pyridoxal-5′-phosphate-dependent decarboxylases, which includes decarboxylases that operate on several different substrates^2^. Two GAD proteins found in vertebrate species, GAD67 and GAD65, are encoded by the paralogous genes *GAD1* and *GAD2*, respectively^3^. While both GADs synthesize GABA and are co-expressed in most vertebrate GABAergic neurons, GAD1 synthesizes cytoplasmic GABA that is used for extrasynaptic and metabolic purposes and GAD2 regulates the vesicular pool for release^4^,^5^,^6^. Nevertheless, *GAD1* and *GAD2* sequences are highly similar to each other, and they share a common intron-exon organization, indicating a common origin^7^.

The evolutionary history of GAD genes is long and diverse. Genes with homology to *GAD* arose before the evolution of eukaryotes^8^. Genes encoding GAD are found, for example, in *Escherichia coli*^9^, *Saccharomyces cerevisiae*^10^, *Drosophila melanogaster*^11^, and *Caenorhabditis elegans*^12^. Furthermore, GABA signaling via membrane receptors elicits hyperpolarization in plants as well as mammals, suggesting conserved or convergent roles for the product of GAD enzymatic activity^13^. In most vertebrate species, only two GAD genes have been described. Another gene in the GAD family, GAD-like 1 (*GADL1*) resembles *GAD1* and *GAD2* in sequence, but is expressed in mouse skeletal muscles and kidney rather than in the brain^14^. There have also been some hints of greater diversity in vertebrate *GAD* genes.

In addition to the teleost *gad1* and *gad2* genes, a third gene, *gad3*, was found in brain cDNA of the abyssal grenadier (*Coryphaenoides (Nematonurus) armatus*), a benthic teleost fish^15^. A similar *gad3* sequence was subsequently identified in the brain cDNA of goldfish (*Carassius auratus*)^16^. The sequences of goldfish and abyssal grenadier *gad3* are clearly related to *gad1a, gad1b,* and *gad2* sequences, but their evolutionary history remained unknown^16^. Furthermore, no *gad3* genes were reported in any species other than grenadier and goldfish. This absence remained an anomaly, since the goldfish (order Cypriniformes), is very distantly related to the abyssal grenadier (order Gadiformes). Recent teleost phylogenies indicate that the Ostariophysians, of which Cypriniformes including goldfish are members, diverged from the Euteleosts, which include the abyssal grenadier, over 250 million years ago^17^. Thus, the conservation of a *gad3* gene in these two divergent species suggested that *gad3* was present in an early teleost ancestor. Because the teleost lineage is known to have experienced a whole-genome duplication early in its evolution, one reasonable possibility could therefore have been that *gad3* was a teleost-specific *gad* paralog^18^,^19^,^20^,^21^.

Since the original identification of *gad3* from teleost brain cDNA, many comparative genomic resources have become available. The sequencing of teleost and other vertebrate genomes has been accompanied by the development of databases and software for analyzing the conservation of genes. Sarcopterygii species with sequenced genomes include primitive fishes, e.g. elephant shark^22^ and coelacanth^23^, as well as tetrapods, e.g. chicken^24^, dog^25^, human^26^, Tasmanian devil^27^, Chinese softshelled turtle^28^, and Xenopus^29^. Actinopterygii species with sequenced genomes include the spotted gar^30^ as well as teleosts like fugu^19^, medaka^31^, tilapia^32^, and zebrafish^33^.

We used recently generated genomic resources to ask whether *gad3* is present and expressed in species other than the goldfish and abyssal grenadier. Our results revealed a surprisingly broad conservation of *GAD3* in mammals, reptiles, birds, and amphibians, as well as fishes.

## Methods

Throughout this paper, we use standard gene nomenclature. For fishes, gene symbols are lowercase and italicized and protein symbols are capitalized. For other vertebrates, human conventions are used: gene symbols in all capitals and italicized, protein symbols in all capitals.

Vertebrate sequence data (Table 1) for *GAD1, GAD2*, and *GAD3* homolog transcripts were downloaded from Ensembl genomes for the following species: chicken (*Gallus gallus*), coelacanth, fugu, human, medaka, spotted gar, Tasmanian devil, tilapia, Chinese softshell turtle, xenopus, zebrafish^34^. Transcript DNA sequences for elephant shark were retrieved from the elephant shark Ensembl server. Transcript cDNA sequences for grenadier were retrieved from NCBI^15^,^35^. The spotted gar genome shares extensive similarity with both tetrapod and teleost genomes, so we chose to focus on this species for sequence alignment, phylogenetics, and intron/exon structure comparisons^30^. Additionally, we obtained sequences for fruitfly (*Drosophila melanogaster*) *GAD1*: NM_079190, sea urchin (*Strongylocentrotus purpuratus*) *GAD*: XM_779763, amphioxus (*Branchiostoma floridae*) *GAD*: XP_002592141, and tunicate (*Ciona intestinalis*) *GAD*: ENSCINT00000004013.

In addition to the species listed in Table 1, we identified several other tetrapod species with *GAD3* genes. These included: Orangutan: ENSPPYG00000009199, Rhesus: ENSMMUG00000001554, Rabbit: ENSOCUG00000022124, Horse: ENSECAG00000009017, Platypus: ENSOANG00000002106, Lizard: ENSACAG00000008555.

### Sequence Alignment

Both DNA and amino acid sequences were aligned using MAFFT v7.017^36^,^37^ (by translation alignment for CDS sequences); algorithm E-INS-I; scoring matrix: BLOSUM62; gap open penalty: 1.53; offset value: 0.

### Model Testing

MEGA 6 software was used to compare 24 DNA evolution models for the aligned GAD CDS sequences^38^. A generalized time-reversible plus gamma (GTR + G + I) model had the lowest BIC score (Bayesian Information Criterion) and AICc value (Akaike Information Criterion, corrected), so it was used for subsequent phylogenetic analyses. In this model, non-uniformity of evolutionary rates among sites is modeled by estimating a discrete Gamma distribution (+G) of rates and by assuming that certain sites are evolutionarily invariable (+I).

### Bayesian Phylogenetic Inference

MrBayes 3.2.6^39^ was used to infer phylogenetic relationships between GAD homologs based on aligned nucleotide CDS sequences, and was accessed via the CIPRES web portal^40^. In MrBayes, the GTR + G + I model of evolution was used; with default settings except for the following specified parameters: nruns(number of runs) = 2; ngen(number of generations) = 1000000; samplefreq = 500; nchain(number of chains) = 8; temp(chain heating temperature) = 0.1; savebrlens = yes; burninfrac(fraction of initial generations discarded) = 0.25.

Diagnostics of the MCMC sampling were carried out using Tracer v1.6 (http://tree.bio.ed.ac.uk/software/tracer/). The effective sample size (ESS) for each parameter was >300 for each run, allowing adequate sampling of the Markov chain.

The tree file generated using MrBayes was visualized using FigTree v1.4.2 (http://tree.bio.ed.ac.uk/software/figtree/).

### Synteny

Gene synteny for *GAD3* genes was compared to the syntenic region near *GAD3* using Genomicus^41^. Orangutan was used as a reference species for cross-species synteny and protein similarity to highlight conservation of synteny and GAD3 protein sequence in vertebrates despite the absence of *GAD3* in some hominids. For comparing primate synteny, simiiformes (last common ancestor of simians) was used as the reference taxon. Synteny data, protein similarity, and species images were downloaded from Genomicus.

## Results

We found previously uncharacterized *GAD3* genes in many vertebrate genomes, including diverse fishes and tetrapods. A teleost *gad3* transcript was found in a transcriptome library generated from testis tissue from *Astatotilapia burtoni*: (>comp56037_c0_seq1_indA_testis). Zebrafish *gad3* has been previously referred to with the identifier *zgc:163121*. Interestingly, *GAD3* had already been annotated in the *Xenopus tropicalis* genome as *GAD1.2*. It appears, however, that it has not yet been studied in *Xenopus*.

### Sequence Similarity

**Figure 1:**
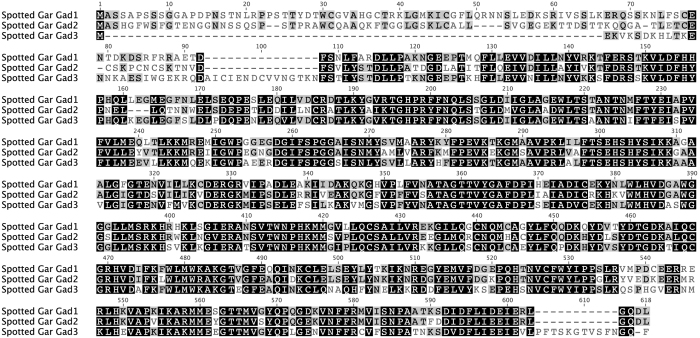
Alignment of predicted protein sequences translated from spotted gar (*Lepisosteus oculatus*) GAD genes. Black: amino acids similar in all three sequences; Gray: similar to one corresponding residue; White: not similar to either corresponding residue. Sequence similarity was calculated using BLOSUM62 matrix with threshold = 1.

Gad3 predicted protein sequence from spotted gar (*Lepisosteus oculatus*) is more similar to Gad1 (Pairwise Identity: 60.5%) than to Gad2 (Pairwise Identity: 53.9%) (Fig. 1). Gad1 and Gad2 share 67.1% pairwise identity. The N-terminal domain, which is quite variable between Gad1 and Gad2, is truncated and highly divergent in Gad3. The N-terminal 92 amino acids (aa) of Gad1 align to the N-terminal 84 aa of Gad2. Gad3 has 42 aa aligning in this range, only 27 of which align to Gad1 and Gad2 sequence (with a 15 aa gap). These 27 aa of Gad3 have: 23.1% pairwise identity with Gad1, 11.5% pairwise identity with Gad2.

### Phylogeny

**Figure 2:**
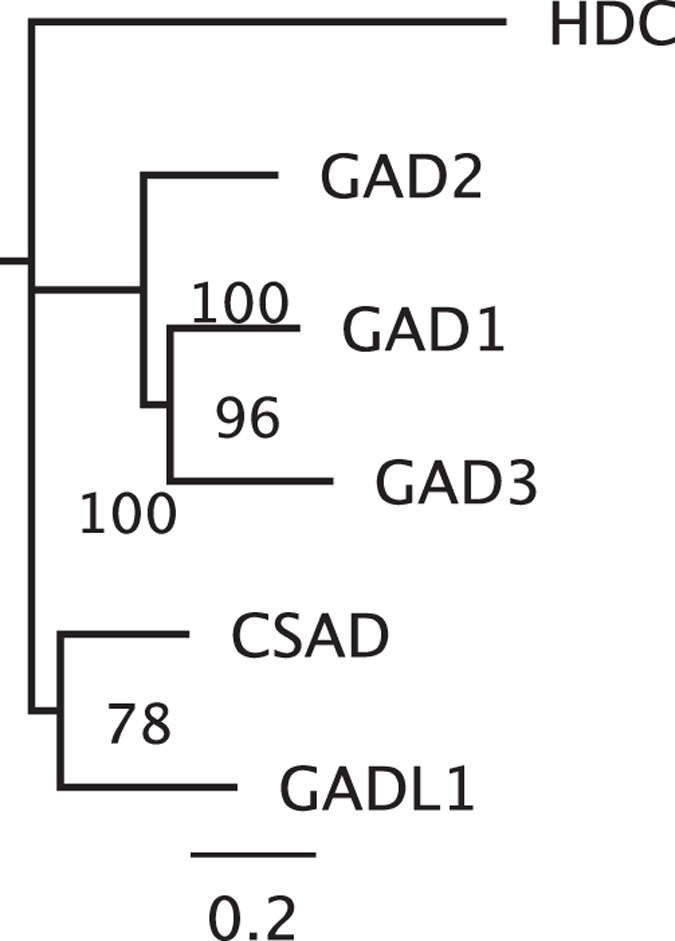
GAD3 is closely related to GAD1 and GAD2, and more distantly related to other members of the PLP-dependent decarboxylase gene family. A phylogenetic tree of aligned PLP-dependent decarboxylase amino acid sequences from the spotted gar was generated using neighbor-joining and 2000 bootstrap iterations. Percent bootstrap support for nodes are shown. The scale bar (bottom) indicates substitutions per site.

Pyridoxal 5′-phosphate (PLP)-dependent decarboxylase genes include Glutamate Decarboxylase-Like 1 (*GADL1*), Cysteine Sulfinic Acid Decarboxylase (*CSAD*), and histidine decarboxylase (*HDC*), in addition to GADs. Therefore, we tested the phylogenetic relationship of *GAD3* to other genes in this group, using *HDC* as an outgroup for *GAD, GADL1,* and *CSAD* genes^42^. A neighbor-joining tree of aligned predicted amino acid sequences from the spotted gar (*Lepisoteus oculatus*) place *gad3* most closely related to the *gad1*/*gad2* clade (Fig. 2).

**Figure 3:**
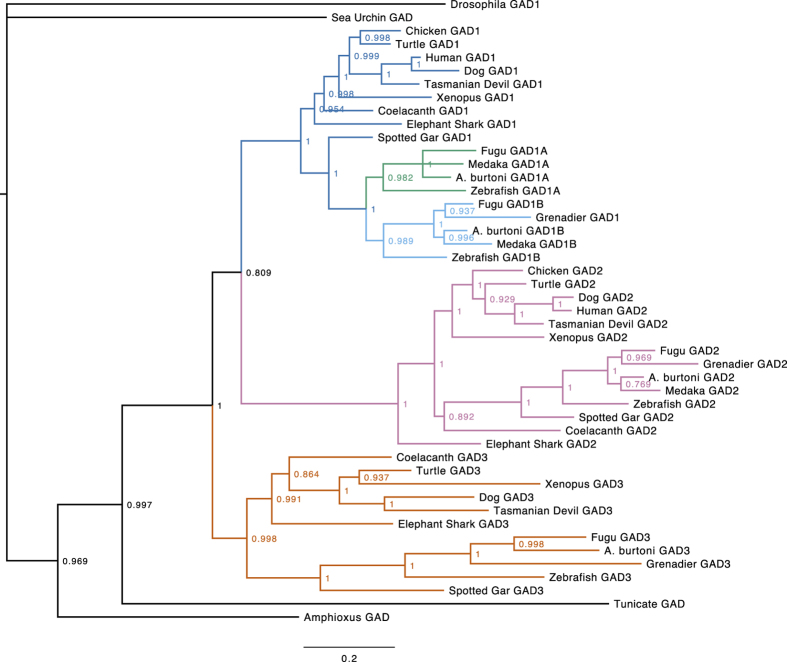
Phylogenetic tree of *GAD1, GAD2*, and *GAD3* nucleotide sequences. This consensus tree was generated using MrBayes with *Drosophila melanogaster GAD1* as the outgroup. Nodes are labeled with posterior probabilities. Distinct gene lineages are indicated by colors. The scale bar (bottom) indicates substitutions per site.

A phylogenetic tree of vertebrate *GAD1, GAD2*, and *GAD3* nucleotide coding sequences was generated using MrBayes (Fig. 3). In insects, *GAD1* is the single homolog of vertebrate GAD genes (insect *GAD2* is homologous to vertebrate *CSAD* and *GADL1*). Therefore we chose *Drosophila melanogaster GAD1* as the outgroup for the vertebrate and deuterostome GAD genes.

### Exon-intron Structure

**Figure 4:**
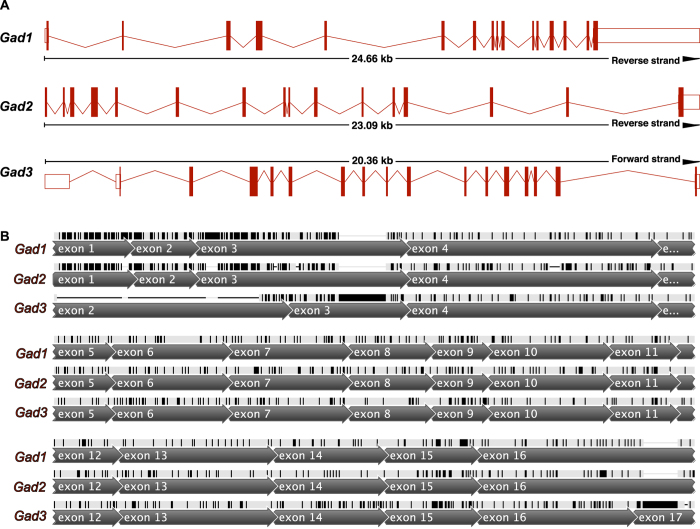
Shared exon-intron structure of GAD genes from spotted gar (*Lepisosteus oculatus*). (**A**) Maps of the three gad genes showing 16 exons in *gad1* and *gad2*, and 17 exons in *gad3*. The direction of transcription is from left to right. Genomic distance spanned and strand on which the gene is located are indicated for each gene. (**B**) Aligned spotted gar *gad1, gad2*, and *gad3* CDS regions annotated with position of exons. Bases colored black indicate disagreements with the consensus sequence of the three gad genes.

Spotted gar *gad1* and *gad2* each have 16 exons (Fig. 4). Spotted gar *gad3* has 17 exons. While both *gad1* and *gad2* have coding sequence beginning in exon 1, *gad3* coding sequence (CDS) begins in the second exon (exon 2). The predicted coding sequence of *gad3* has a gap (does not align) with the 5′ CDS sequence found in *gad1* and *gad2* exon 1, exon 2, and part of exon 3. The 3′ portion of the *gad3* CDS is included on exon 17, while *gad1* and *gad2* stop codons are found in exon 16.

Aside from these differences, *gad3* exon structure is largely similar to *gad1* and *gad2*. All of the exon junctions from exon 3 to exon 16 are in identical locations for all three gad genes. In our alignment of the three *gad* genes, the only gaps introduced in *gad3* are located in exon 2 and exon 17.

### Synteny

**Figure 5:**
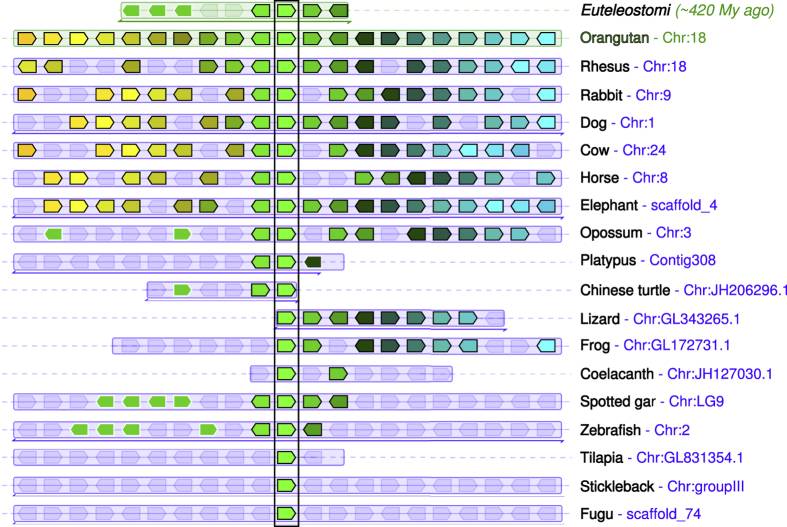
*GAD3* genes are found in a conserved syntenic region in vertebrates. Compared to orangutan *GAD3* for reference, other species including representatives of mammals, other tetrapods, and Euteleostomi (bony vertebrates) in general have similar chromosomal positions of *GAD3* genes. The central green pentagons (surrounded by a vertical rectangle) represent the *GAD3* genes. For each species, *GAD3* and 10 flanking genes on each side are represented by colored pentagons. The pentagons point in the direction of transcription, and each color identifies a set of orthologous genes. To the right of the figure, both species and chromosome are indicated for each *GAD3* ortholog. Figure modified from Genomicus PhyloView output^41^.

In spotted gar, *gad1* and *gad2* are located adjacent to the myosin genes *myo3b* and *myo3a*, respectively. Similarly, in humans *GAD1* is located near *MYO3B* on chromosome 2, and *GAD2* is located adjacent to *MYO3A* on chromosome 10. On the other hand, *gad3* is not located near a myosin gene in the genome of spotted gar. The genes located adjacent to spotted gar *gad3* are *mc4r* and *cdh20*. This syntenic block of genes is conserved across many vertebrate species, and represents the inferred ancestral state of the bony vertebrates (euteleostomi) (Fig. 5).

### GAD3 Conservation and Gene Loss

**Figure 6:**
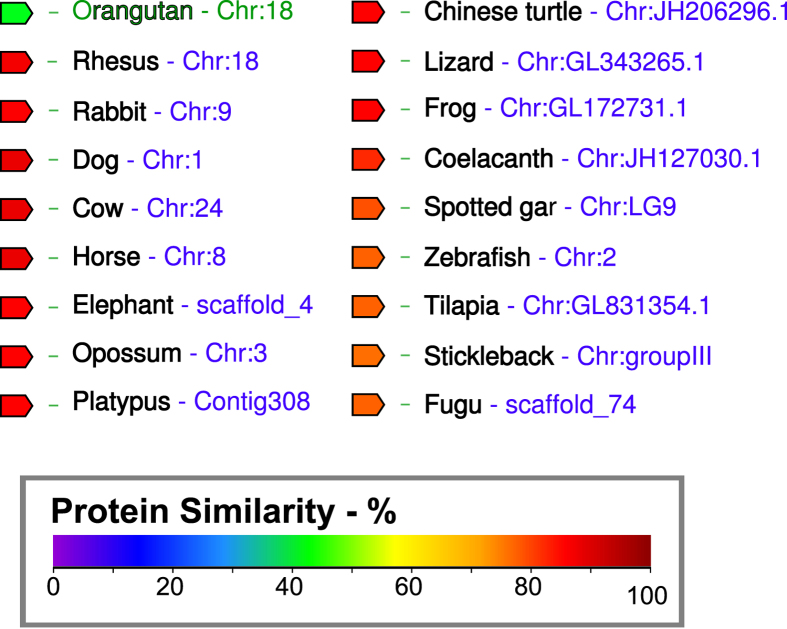
GAD3 protein sequence is highly conserved across Euteleostomi. Predicted protein sequences of GAD3 orthologs were compared using Genomicus^41^. The degree of similarity to the reference sequence (orangutan, in green) is indicated by the color of the block, according to the scale shown at bottom.

**Figure 7:**
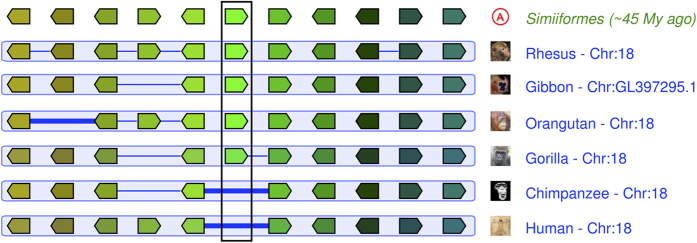
*GAD3* is lost in hominids. The central green pentagons (surrounded by a vertical rectangle) represent the *GAD3* genes. Human and chimpanzee genomes have no functional gene at the location corresponding to *GAD3* in other primates. Humans have a pseudogene at this location. A thick blue line between two genes indicates a “gap”, i.e. a gene lost relative to the ancestral Simiiformes genome. A thin blue line between two genes indicates a “break” in the continuity of the alignment, i.e. a gene added or lost relative to the ancestral Simiiformes genome. Figure modified from Genomicus AlignView^41^, which relies on data and images from Ensembl^34^.

GAD3 predicted protein sequence is highly conserved in diverse vertebrate genomes (Fig. 6). Yet primates appear to have experienced varying degrees of gene loss at the *GAD3* locus (Fig. 7). We identified a human transcript (ENST00000592477) with homology to *GAD3* (Table 1), derived from a pseudogene located in the human genome in the conserved *GAD3* syntenic position between *MC4R* and *CDH20* (Fig. 7). Similarly, gorilla (*Gorilla gorilla*) *GAD3* is annotated as a pseudogene in Ensembl (ENSGGOG00000027455). Although macaque (*Macaca mulatta*) and orangutan (*Pongo pygmaeus*) predicted GAD3 protein sequences share relatively high pairwise identity (84.5%), the *GAD3* genes in these two species appear to have large insertions (or deletions) in their predicted coding sequence.

## Discussion

We identified a novel glutamate decarboxylase homolog, *GAD3*, found in many vertebrate genomes. We provide phylogenetic and intron/exon structural evidence that *GAD3* is an ancient paralog of *GAD1* and *GAD2*. The conserved chromosomal synteny of *GAD3* in vertebrates supports an ancient origin for this gene. Surprisingly, *GAD3* was lost in the hominid lineage. Taken together, the phylogenetic analyses, comparisons of gene structure, and synteny data suggest that *GAD3* arose via gene duplication of a protovertebrate *GAD* homolog, likely before the duplication of another paralog which gave rise to *GAD1* and *GAD2*.

### GAD3 Evolution

Although our data do not rule out the possibility of a local duplication that gave rise to *GAD3*, they are consistent with an origin of *GAD3* in an early vertebrate via whole-genome duplication. Whole-genome duplication is thought to have played a major role in early vertebrate evolution^43^,^44^,^45^. Following genome duplication, these duplicated gene pairs (ohnologs) experienced a range of outcomes including non-functionalization, sub-functionalization, and neo-functionalization^46^,^47^. Sub-functionalization may happen via protein changes^48^ or via regulatory element loss^49^ in which ancestral expression domains are differentially lost in different genes^50^. For example, recent evidence indicates that duplication of a corticotropin-releasing hormone (*CRH*) gene in an early vertebrate led to a broadly expressed *CRH1* and a *CRH2* with expression restricted to a single hindbrain nucleus^51^. Like our recent analyses of CRH genes, the discovery of *GAD3* as a conserved vertebrate gene relied on freely available genomic resources, pointing to the likelihood that many gene families have unannotated homologs remaining to be found in sequenced genomes^51^,^52^.

### GAD3 Function

GAD3 is phylogenetically closer to GAD1 and GAD2 than to GADL1, but nonetheless it is possible that its enzymatic functions differ from those of GAD1 and GAD2. The absence of much of the N-terminal region, which regulates intracellular localization of GAD1 and GAD2 proteins, suggests that GAD3 protein may have different localization^5^.

Little is known regarding the function of GADL1 enzyme, though polymorphisms are linked to differential response to lithium treatment for bipolar disorder^53^. Mammalian GADL1 does not appear to have glutamate decarboxylase activity, despite its name. Instead, it catalyzes the decarboxylation of aspartate, cysteine sulfinic acid, and cysteic acid to β-alanine, hypotaurine, and taurine, respectively^14^. Recently, GADL1 and CSAD were found to have preference for cysteine sulfinic acid as a substrate^42^. Future studies of GAD3 biochemical substrates will be necessary to address the possibility of substrates other than glutamate.

Intriguingly, zebrafish *gad3* (referred to as *zgc163121*) mRNA expression was significantly downregulated by treatment with dexamethasone, a glucocorticoid agonist, in 25hpf larval zebrafish, as measured by microarray and qPCR^54^. In the deep-sea fish in which *gad3* was first described, the armed grenadier, *Coryphaenoides (Nematonurus*) *armatus, gad2* mRNA levels were found to be expressed in the brain in a sexually dimorphic manner, i.e. higher in male hypothalamus than in female, but no differences were found in *gad3* levels^35^. In the goldfish, however, *gad3* mRNA levels in the telencephalon were highest in sexually mature fish of both sexes during the breeding period^55^. Since the specific role of *gad3* is unknown in any taxa, the full range of factors that regulate *gad3* expression in the brain, and potentially elsewhere, awaits further investigation.

### Loss in hominids

The loss of *GAD3* in both chimpanzees and humans appears to have been preceded by changes to *GAD3* sequences in other hominids. Predicted gorilla, orangutan, and gibbon *GAD3* transcripts appear to be truncated relative to fish *gad3* sequence, but it may be that not all the exons in these sequences are fully annotated in Ensembl. Glutamate metabolic pathways appear to have been under positive selection in hominids, as seen for example in the origin of glutamate dehydrogenase 2 (*GLUD2*) by retroposition of *GLUD1*^56^.

Gene losses have played major roles in human evolution^57^. For example, loss of L-gulonolactone oxidase (*GULO*) makes humans and other Haplorhini susceptible to scurvy, a vitamin C deficiency. Despite conferring this disadvantage, GULO gene loss has occurred in multiple mammalian lineages, including guinea pigs^58^ and some bats^59^.

Hominids are not the only lineage that has lost *GAD3*. Rodent genomes, including mice, rats, and squirrels also appear to be missing *GAD3* homologs. The absence of *GAD3* in both humans and mice likely explains why this gene was not discovered sooner, since many investigators choose to focus on these two species.

## Additional Information

**How to cite this article**: Grone, B. P. and Maruska, K. P. Three Distinct Glutamate Decarboxylase Genes in Vertebrates. *Sci. Rep.* **6**, 30507; doi: 10.1038/srep30507 (2016).

## Figures and Tables

**Table 1:** Vertebrate *GAD1, GAD2*, and *GAD3* transcript sequence IDs.

Species	Scientific Name	*GAD1*	*GAD2*	*GAD3*
Chicken	*Gallus gallus*	ENSGALT00000043162	ENSGALT00000012268	
Burton’s mouthbrooder	*Astatotilapia burtoni*	Gad1a: XM_014332345 Gad1b: XM_014340384	XM_005932121	XM_005950266
Coelacanth	*Latimeria chalumnae*	ENSLACT00000014577	ENSLACT00000011268	ENSLACT00000005682
Dog	*Canis familiaris*	ENSCAFT00000049584	ENSCAFT00000006929	ENSCAFT00000000144
Elephant shark	*Callorhinchus millii*	SINCAMT00000000719	SINCAMT00000011054	SINCAMT00000005039
Fugu	*Takifugu rubripes*	Gad1a:ENSTRUT00000045798 Gad1b:ENSTRUT00000020549	ENSTRUT00000024751	ENSTRUT00000021119
Abyssal grenadier	*Coryphaenoides armatus*	AF043268	AF043267	AF043269
Human	*Homo sapiens*	ENST00000358196	ENST00000376261	ENST00000592477*
Medaka	*Oryzias latipes*	Gad1a:ENSORLT00000021605 Gad1b:ENSORLT00000011550	ENSORLT00000016248	
Spotted Gar	*Lepisosteus oculatus*	ENSLOCT00000009532	ENSLOCT00000009370	ENSLOCT00000015874
Tasmanian Devil	*Sarcophilus harrisii*	ENSSHAT00000013524	ENSSHAT00000015741	ENSSHAT00000004379
Nile tilapia	*Oreochromis niloticus*	Gad1a:ENSONIT00000011023 Gad1b:ENSONIT00000023558	ENSONIT00000008095	ENSONIT00000008040
Chinese softshelled turtle	*Pelodiscus sinensis*	ENSPSIT00000002371	ENSPSIT00000019780	ENSPSIT00000019627
Xenopus	*Xenopus tropicalis*	ENSXETT00000040862	ENSXETT00000040531	ENSXETT00000012900
Zebrafish	*Danio rerio*	Gad1a:ENSDART00000140425 Gad1b:ENSDART00000003008	ENSDART00000021609	ENSDART00000109561

*pseudogene transcript sequence.
